# The effect of left atrial wall thickness and pulmonary vein sizes on the acute procedural success of atrial fibrillation ablation

**DOI:** 10.1007/s10554-022-02533-y

**Published:** 2022-02-09

**Authors:** Melinda Boussoussou, Bálint Szilveszter, Borbála Vattay, Márton Kolossváry, Milán Vecsey-Nagy, Zoltán Salló, Gábor Orbán, Perge Péter, Piros Katalin, Nagy Klaudia Vivien, Osztheimer István, Pál Maurovich-Horvat, Béla Merkely, László Gellér, Nándor Szegedi

**Affiliations:** 1 Semmelweis University Heart and Vascular Center, Városmajor utca 68., Budapest, 1122 Hungary; 2 Medical Imaging Centre, Korányi Sándor u. 2., Budapest, 1082 Hungary

**Keywords:** Radiofrequency ablation, Pulmonary vein isolation, Atrial fibrillation, Computed tomography, CLOSE protocol, Left atrial wall thickness

## Abstract

Nowadays, a novel contact-force guided ablation technique is used for enclosing pulmonary veins in patients with atrial fibrillation (AF). We sought to determine whether left atrial (LA) wall thickness (LAWT) and pulmonary vein (PV) dimensions, as assessed by cardiac CT, could influence the success rate of first-pass pulmonary vein isolation (PVI). In a single-center, prospective study, we enrolled consecutive patients with symptomatic, drug-refractory AF who underwent initial radiofrequency catheter ablation using a modified CLOSE protocol. Pre-procedural CT was performed in all cases. Additionally, the diameter and area of the PV orifices were obtained. A total of 1034 LAWT measurements and 376 PV area measurements were performed in 94 patients (mean CHA_2_DS_2_-VASc score 2.1 ± 1.5, mean age 62.4 ± 12.6 years, 39.5% female, 38.3% persistent AF). Mean procedure time was 81.2 ± 19.3 min. Complete isolation of all PVs was achieved in 100% of patients. First-pass isolation rate was 76% and 71% for the right-sided PVs and the left-sided PVs, respectively. No difference was found regarding comorbidities and imaging parameters between those with and without first-pass isolation. LAWT (mean of 11 regions or separately) had no effect on the acute procedural outcome on logistic regression analysis (all p ≥ 0.05). Out of all assessed parameters, only RSPV diameter was associated with a higher rate of successful right-sided first pass isolation (OR 1.01, p = 0.04). Left atrial wall thickness does not have an influence on the acute procedural success of PVI using ablation index and a standardized ablation protocol. RSPV diameter could influence the probability of right sided first-pass isolation.

## Introduction

Atrial fibrillation (AF) is the most common sustained cardiac arrhythmia [1], with globally increasing prevalence and incidence. Since triggers of AF originate from the pulmonary veins (PVs), pulmonary vein isolation (PVI) became the backbone of the treatment of AF [2, 3]. The durable isolation of the PVs remains challenging; however, new technologies might facilitate achieving better results [4]. One of the most recent catheter ablation strategies is the CLOSE protocol, a contact-force-guided approach using contiguous and optimized radiofrequency lesions to enclose pulmonary veins [5–7]. The CLOSE protocol and modified CLOSE protocols [8] were shown to provide excellent procedural outcomes in recent studies evaluating the safety and 1-year single-procedural freedom from AF. Lesion contiguity and consistency are substantial factors of acute procedural success in such procedures. Moreover, the contiguous and durable lesion set might be associated with a higher chance of long-term arrhythmia-free survival [9].

The importance of cardiac CT before AF ablation is unquestionable, as it helps both to the plan of the procedure and to the selection of optimal patients for ablation [10–12]. Left atrial (LA) wall thickness (LAWT) and PV anatomy, assessed by cardiac CT, might influence the efficacy of radiofrequency catheter ablation [9, 13, 14]. It has been suggested that greater local atrial wall thickness could lead to reconnected PVs and thus the CLOSE protocol might need further modifications to create proper LA lesions. However, less advanced ablation strategies were used in those studies, therefore former results may not apply for the latest ablation techniques.

We hypothesised that using a novel, modified CLOSE protocol proper isolation of the PVs is achievable even in patients with larger LAWT. First-pass isolation is a valuable marker of PVI’s acute procedural success [15, 16]. The effects of PV anatomy and LAWT on successful first-pass isolation has not yet been investigated. Therefore, our current study aimed to determine the relationship between the acute procedural success assessed by the presence of first-pass isolation and LAWT, measured by cardiac CT. Moreover, we assessed the potential role of PV anatomy in the rate of first-pass isolation.

## Methods

### Patient population

**Fig. 1:**
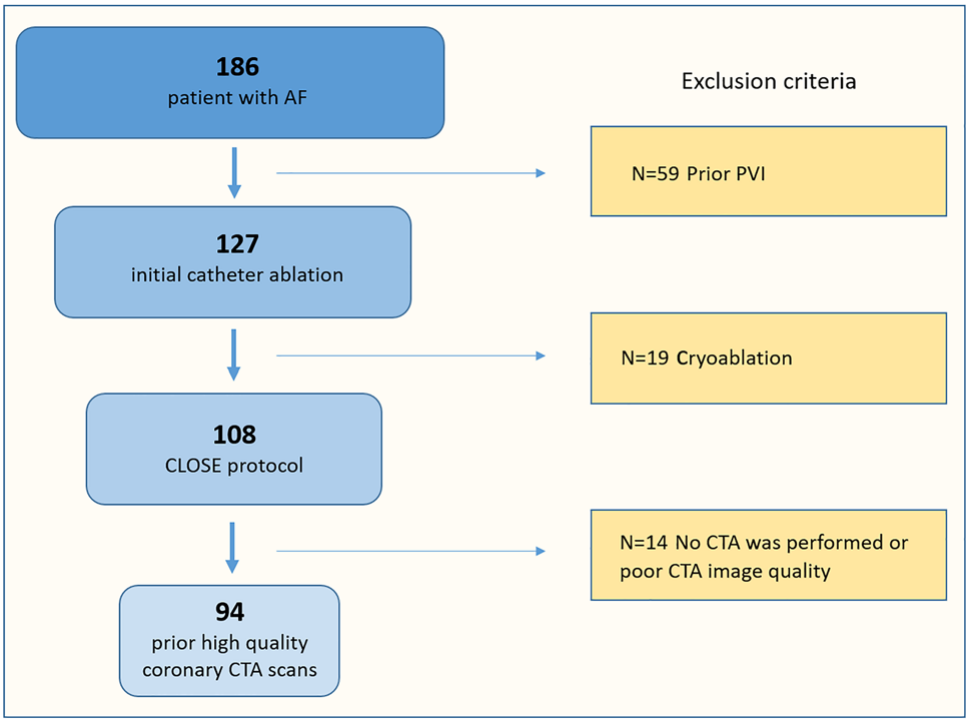
Patients with atrial fibrillation were enrolled in the study population, between the time interval of 2019 January to 2020 December who underwent PVI using the CLOSE protocol. After excluding cases with cryoablation, prior PVI and suboptimal CT image quality a total of 94 patients were analysed. *AF* atrial fibrillation, *PVI* pulmonary vein isolation

In our single-center, prospective, observational cohort study, 186 consecutive patients with symptomatic drug-refractory AF were screened who underwent radiofrequency ablation between January of 2019 and September of 2020. Exclusion criteria were previous catheter ablation procedure, absence of pre-procedural LA cardiac CT or poor cardiac CT image quality (Consort diagram: Fig. 1). Overall, we analyzed a total of 94 patients who met all inclusion and exclusion criteria.

All patients agreed to the pre-procedural imaging and the ablation procedure and provided written consent to data retrieval and analysis. The study protocol was reviewed and approved by the institutional review board and was in accordance with the declarations of Helsinki.

### CT imaging of the left atrium and the pulmonary veins

All patients underwent contrast-enhanced multi-detector CT imaging before the catheter ablation procedure to determine the PV and LA anatomy. Cardiac CT scans were performed on a 256-slice scanner (Brilliance iCT 256, Philips Healthcare, Best, The Netherlands) with prospective ECG-triggered axial acquisition mode during inspiratory breath hold and arm raised position. For proper heart-rate control, oral or intravenous beta-blocker was administered before the CT scans in patients with a heart rate above 65 beats per minute. In patients with a heart rate of less than 75 beats per minute, mid-diastolic triggering was applied with 3–5% padding (73–83% of the R–R interval), and in those with ≥ 75 beats per minute, systolic triggering was chosen (35–45% of the R–R interval). Depending on body mass index, 100–120 kV tube voltage and 200–300 mAs tube current were used. Image acquisition was performed with 270-ms gantry rotation time and 128 × 0.625-mm detector collimation. Intra venous iodinated contrast agent (80–100 ml Iomeron 400, Bracco Imaging Ltd.) was administered at a flow rate of 4.5–5.5 ml/s via 18-gauge catheter from antecubital vein access using a four-phasic contrast protocol as described elsewhere [17]. XCC convolution kernel, and iDose level 5 iterative reconstruction were used. CT data sets were reconstructed with 0.8-mm slice thickness and 0.4-mm increment [18].

### Image analysis

**Fig. 2:**
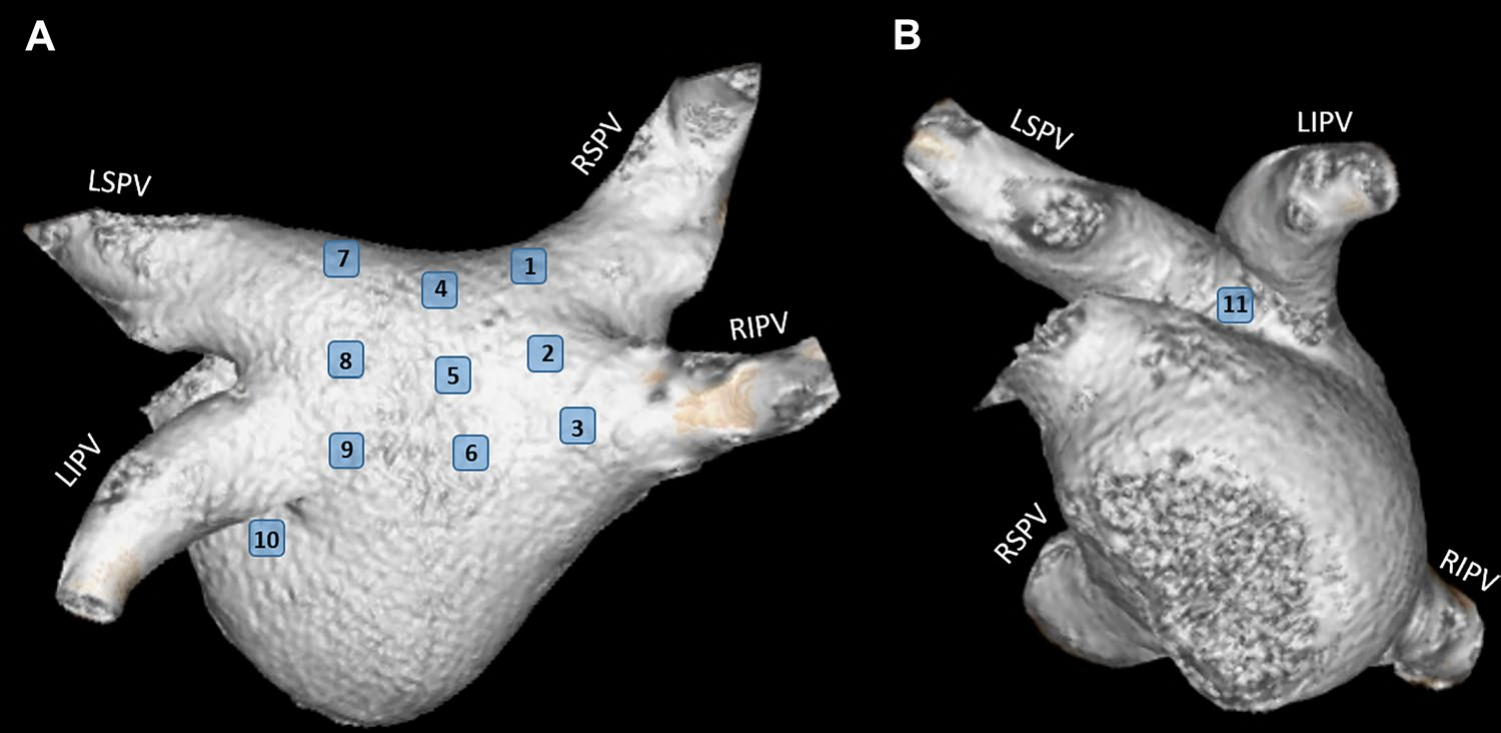
Volume rendered 3D CT images demonstrate the 11 locations where the wall thickness was measured in the left atrium (modified after Hayashi et al. [24]). On **A** the LA is in a posterior view whereas **B** represents the left lateral view of the LA. The numbers show the following left atrial areas: 1: right roof. 2: right mid-posterior, 3: right infero-posterior, 4: middle part of the roof, 5: middle part of the mid-posterior, 6: middle part of the infero-posterior, 7: left of the roof, 8: left of the mid-posterior, 9: left of the infero-posterior, 10: mitral isthmus, 11: left lateral ridge. *LA* left atrium, *LIPV* left inferior pulmonary vein, *LSPV* left superior pulmonary vein, *RIPV* right inferior pulmonary vein, *RSPV* right superior pulmonary vein

**Fig. 3:**
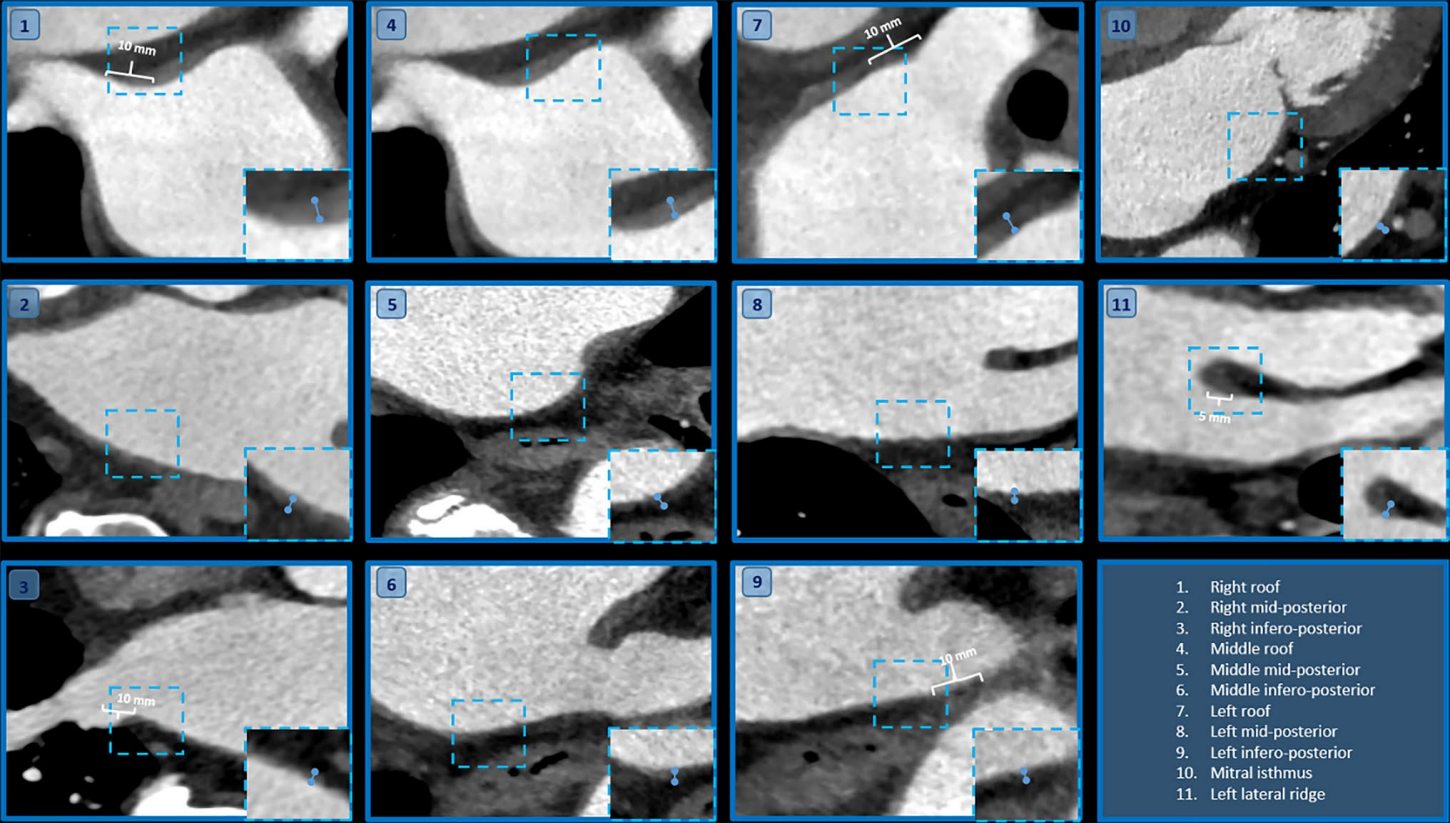
Left atrial wall thickness (LAWT) was measured at 11 sites based on Hayashi et al. as demonstrated on the CT images. The numbers indicate the same locations for LAWT assessment as illustrated in Fig. 2. The dashed squares show the area of interest which are enlarged in the right-hand corner of every image. The blue line between the two dots represents the size of the measured LAWT. The roof and the infero-posterior wall thickness (Number 1, 3, 7 and 9) were assessed at 10 mm distance from the junction of the left atrium and pulmonary vein. LAWT at the ridge was measured at the center of the left superior PV (5 mm from the LA wall). *LA* left atrium, *LAWT* left atrial wall thickness, *PV* pulmonary vein

The measurements of the LAWT and the PVs were carried out by utilizing a commercially available software (Philips IntelliSpace Portal v.6.2, Philips Healthcare). The maximum wall thickness areas were assessed in 11 separate LA locations, including the right, middle and left part of the roof, mid-posterior and infero-posterior regions. Representative images of each location are depicted on Fig. 2. These areas of interest were considered the most commonly targeted locations during catheter ablation procedures of AF [19–25]. In addition, the wall thickness at the left lateral ridge and mitral isthmus were evaluated based on Hayashietal et al. [24]. To measure the roof thickness of the LA, an oblique coronal plane was acquired parallel to the superior PV or posterior wall (Number 1, 4, 7 in Figs. 2 and 3), whereas to measure the mid-posterior and infero-posterior wall (Number 2, 5, 8, 3, 6, 9 in Figs. 2 and 3) an oblique axial plane perpendicular to the posterior LA wall was acquired. The LAWT at the right and left roof and right and left infero-posterior areas were assessed 10 mm away from the LA–PV connection. The wall thickness at the mitral isthmus was measured by obtaining an axial plane that corresponds to Number 10 in Figs. 2 and 3. The LA ridge wall thickness was measured 5 mm inside the center of the left superior PV with an oblique perpendicular plane view to the superior left lateral ridge (Number 11 in Figs. 2 and 3). We calculated mean and maximal LAWT for the left (Number 7–9) and right side (Number 1–3) at the thickest portion of the given segment and for all measured segments.

**Fig. 4:**
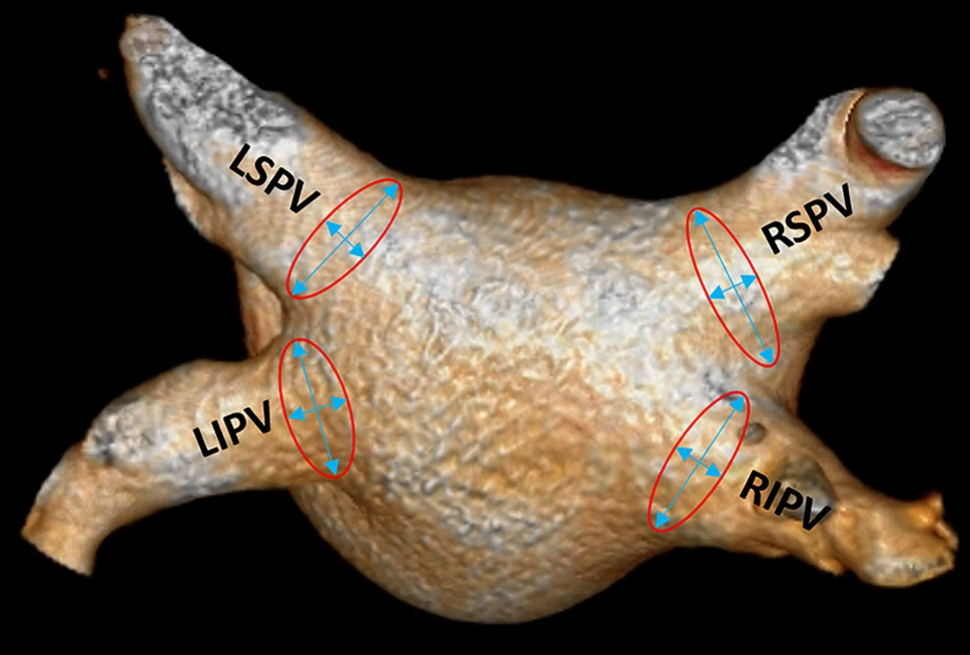
Volume-rendered images show a typical pulmonary vein anatomy with left inferior and superior pulmonary veins and right inferior and superior pulmonary veins. The red circles represent the area of the ostium of each PVs while the blue arrows the maximum and minimum diameters of the orifices. *LIPV* left inferior pulmonary vein, *LSPV* left superior pulmonary vein, *RIPV* right inferior pulmonary vein, *RSPV* right superior pulmonary vein

The PV anatomy and diameter were also analyzed for all patients on the contrast-enhanced cardiac CT images. A normal PV anatomy was defined as the presence of four distinct PVs (e.g. left superior, left inferior, right superior and right inferior pulmonary veins) (Fig. 4). Left or right common trunk was defined when the superior and inferior PVs were connected/fused into one common ostium. First, we selected a given PV orifice and adjusted centerlines manually. The orifices were defined at the angle where the veins departed from the curvature of the LA [26] then we measured the areas and effective diameters perpendicular to the vessel wall based on the maximum and minimum diameter of the orifices [13]. In case of a left or right common trunk the measurements were carried out in the common ostium.

### Ablation procedure

**Fig. 5:**
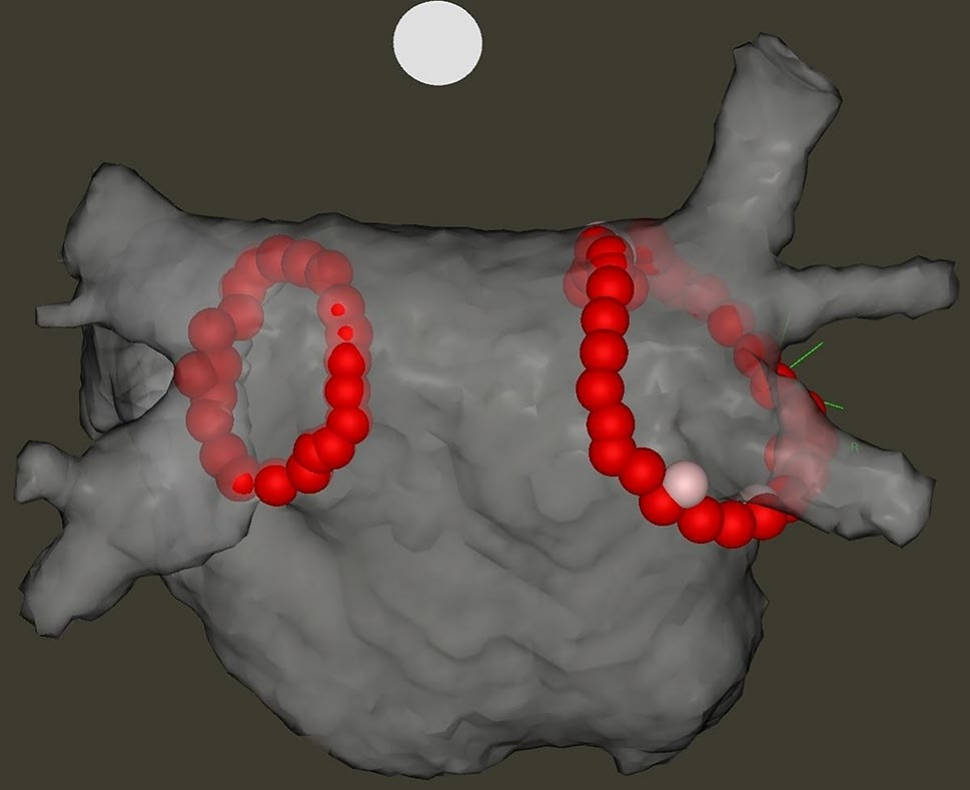
Electroanatomical map of the left atrium after pulmonary vein isolation performed with the modified CLOSE protocol (postero-anterior view). All red ablation tags indicate an ablation index value > 400 on the posterior wall and > 500 on the anterior wall. The inter-tag distance is < 6 mm between all points

Indications for AF ablation procedures and periprocedural anticoagulation were in accordance with the current guideline [27]. PVI was performed with radiofrequency energy, using the point-by-point technique, with the support of the CARTO3 (Biosense Webster, Inc., Baldwin Park, CA, USA) electroanatomical mapping system. The goal of each procedure was the complete electrical isolation of all PVs from the LA with circumferential, contiguous ablation lines. First, a fast anatomical map of the LA was created with a multipolar mapping catheter (Lasso® NAV Eco, Biosense Webster, Inc., Baldwin Park, CA, USA). Then, radiofrequency ablations were applied in a point-by-point manner with ThermoCool SmartTouch® (Biosense Webster, Inc., Baldwin Park, CA, USA) ablation catheter through a steerable sheath (Agilis, Abbott). The ablations were guided by the modified CLOSE protocol: inter-lesion distance < 6 mm at all sites, ablation index target value 400 on the posterior wall and 500 on the anterior wall, target contact force 10–40 g (Fig. 5). During ablation, the mapping catheter was placed in the contralateral PVs to blind the operator for the presence or absence of first-pass isolation. After completing the circumferential ablation line around the ipsilateral PVs, the mapping catheter was placed in the ablated PVs and both entrance and exit block were evaluated. Entrance block was defined by the absence of local PV potentials on the mapping catheter placed in the PVs, while exit block was assessed by pacing at multiple sites inside the PVs. First-pass isolation was defined as the presence of both entrance and exit block after finishing the first-pass ablation circle. If PV conduction was still present after finishing the first-pass ablation circle, it was defined as first-pass isolation absent. Of course, in these cases, ablation was continued until bidirectional PV disconnection was achieved. After finishing the ablation on one side, the mapping catheter was left in those PVs and ablation was performed at the other side as well. Again, after finishing the ablation circle at the other side, the mapping catheter was moved to these PVs to assess the entrance and exit block and the presence or absence of first-pass isolation. All PVs were repeatedly evaluated after a 20 min waiting period to assess acute PV reconnection. All patients without complications were discharged the day after the procedure.

### Statistical analysis

Continuous variables are presented as mean and standard deviation, whereas categorical parameters are presented as frequency with percentages. Independent sample t-test was used to compare LAWT between different LA wall territories or for assessing the differences between PVs with or without first pass isolation.

Thereafter logistic regression analysis was used to identify predictors of procedural success regarding the left or the right pulmonary veins. Univariate logistic regression models included anthropometrical parameters, comorbidities, periprocedural data, LA appendage flow as assessed by transeosophagial echocardiography, left atrial volume (LAV), mean and maximum LAWT, PV diameters according to sidedness (left sided LAWT and PV diameters for the ablation of the left side and right sided LAWT and PV diameters for the right side).

Reproducibility of measurements (intra- and inter-observer agreement) was carried out using intraclass correlation coefficient (ICC) in 20 patients by a single reader. All analyses were conducted using STATA v13.0. A two-sided p-value smaller than 0.05 was considered statistically significant.

## Results

Overall, 94 patients were included in the analysis (mean age 62.4 ± 12.6 years, mean body mass index (BMI) 28.1 ± 3.5 kg/m^2^, 39.4% female). The total number of LAWT measurements were 1034. Moreover, 376 pulmonary vein ostium diameters and areas were measured. The baseline characteristics of the study population are summarized in Table 1.

**Table 1:** Patient characteristics

Parameters	Total N = 94	Successful first-pass isolation in all PVs N = 51	Absence of first-pass isolation in at least one PV N = 43	p value
Antropometric data and comoribities				
Age (years), (mean ± SD)	62.4 ± 12.6	62.8 ± 10.3	62.0 ± 15.0	0.06
Female sex, n (%)	37 (39.40)	23 (45.10)	14 (32.60)	0.21
Body mass index (kg/m^2^), (mean ± SD)	28.14 ± 3.49	27.91 ± 3.56	28.44 ± 3.43	0.81
Body surface area (m^2^), (mean ± SD)	2.04 ± 0.19	2.04 ± 0.20	2.06 ± 0.20	0.14
Diabetes, n (%)	14(14.90)	5 (9.80)	0 (0.0)	0.06
Hypertension, n (%)	61(64.90)	36 (70.60)	25 (58.10)	0.20
Hyperlipidaemia, n (%)	26 (27.70)	19 (37.30)	7 (16.30)	**0.03**
Prior Stroke/TIA, n (%)	4 (4.30)	2 (3.90)	2 (4.70)	0.86
Persistent AF, n (%)	36 (38.30)	17 (33.30)	19 (44.20)	0.28
CHA_2_DS_2_-VASc score, (mean ± SD)	2.11 ± 1.46	2.03 ± 1.36	2.10 ± 1.57	0.71
Procedural parameters				
LAT (min), (mean ± SD)	61.87 ± 15.63	62.08 ± 17.29	61.62 ± 13.57	0.89
Procedure time (min), (mean ± SD)	81.16 ± 19.29	75.31 ± 14.21	88.44 ± 22.27	**0.01**
Fluoroscopy time (min), (mean ± SD)	214.15 ± 177.28	192.69 ± 172.03	239.18 ± 182.17	0.61
Fluoroscopy dose (mGym2), (mean ± SD)	0.34 ± 1.13	0.22 ± 0.443	0.49 ± 1.61	0.06
Echocardiographic parameters				
Ejection fraction (%), (mean ± SD)	57.90 ± 8.00	58.28 ± 7,57	57.38 ± 8.51	0.63
LAA flow, cm/s	57.08 ± 24.24	57.58 ± 24.19	56.37 ± 24.71	0.40
CT derived parameter				
LAV (ml), (mean ± SD)	101.61 ± 39.82	99.67 ± 35.43	104.02 ± 45.03	0.08

Significant values are marked in bold

*AF* atrial fibrillation, *CI* confidence interval, *LAA flow* left atrial appendage flow, *LAT* left atrial time, *LAV* left atrial volume, *LAWT* left atrial wall thickness, *LIPV* left inferior pulmonary vein, *LSPV* left superior pulmonary vein, *PV diameter* pulmonary vein diameter, *RIPV* right inferior pulmonary vein, *RSPV* right superior pulmonary vein, *SD* standard deviation, *OR* odds ratio, *TIA* transient ischemic attack

### Left atrial measurements

The mean LAWT was 1.35 ± 0.46 mm with a range of 0.2–2.6 mm. Results of CT-based assessment of LAWT are shown in Table 2. The left infero-posterior mean thickness was the lowest with a mean value of 0.83 ± 0.49 mm, and was significantly smaller compared to the right roof, right mid-posterior, right infero-posterior, middle roof, middle mid-posterior and the mitral isthmus and left lateral ridge LA locations (p < 0.01). The left lateral ridge had the largest LAWT, with a mean value of 1.95 ± 0.77 mm and was significantly larger than other LA locations (p < 0.01).

**Table 2:** The left atrial wall thickness values in 11 locations

LA locations	Mean	SD	Standard error	95% CI for mean		Minimum	Maximum
				Lower bound	Upper bound		
1	1.79	0.81	0.08	1.63	1.96	0.20	4.20
2	1.93	0.79	0.08	1.77	2.09	0.10	4.00
3	1.56	0.75	0.07	1.41	1.72	0.10	3.10
4	1.21	0.60	0.06	1.09	1.33	0.10	2.80
5	1.29	0.66	0.06	1.16	1.43	0.20	3.20
6	0.97	0.54	0.05	0.86	1.08	0.10	2.50
7	1.01	0.52	0.05	0.89	1.11	0.10	2.40
8	1.01	0.59	0.06	0.89	1.13	0.20	2.80
9	0.83	0.49	0.05	0.73	0.94	0.10	2.30
10	1.24	0.67	0.06	1.10	1.38	0.10	2.70
11	1.95	0.77	0.08	1.79	2.11	0.20	4.00
Total	1.35	0.76	0.02	1.30	1.39	0.10	4.20

1: right roof, 2: right mid-posterior, 3: right infero-posterior, 4: middle roof, 5: middle mid-posterior, 6: middle infero-posterior, 7: left roof, 8: left mid-posterior, 9: left infero-posterior, 10: mitral isthmus, 11: left lateral ridge

*CI* confidence interval, *LA* left atrium, *SD* standard deviation

Regional differences were assessed by combining several measurement points. We found that LAWT on the right side (roof, mid-posterior, infero-posterior) was significantly larger as compared to the middle (roof, mid-posterior, infero-posterior) and left side (roof, mid-posterior, infero-posterior) (all p < 0.01). The infero-posterior region (right infero-posterior, middle infero-posterior, left infero-posterior) was substantially thinner than the mean middle (right mid-posterior, middle mid-posterior, left mid-posterior) and mean roof total (right roof, middle roof, left roof (p = 0.01 and p = 0.08, respectively). Table 3 summarizes PV diameters and areas.

**Table 3:** CT based assessment of LA-PV parameters

	First-pass on left side	Unsuccessful first-pass on left side	p value	First-pass on right side	Unsuccessful first-pass on right side	p value
PV diameter (mm), (mean ± SD)						
LIPV	17.6 ± 6.0	16.4 ± 4.0	0.31	NA	NA	NA
LSPV	18.6 ± 3.1	17.2 ± 3.9	0.19	NA	NA	NA
RSPV	NA	NA	NA	21.3 ± 3.2	19.9 ± 3.7	**0.04**
RIPV	NA	NA	NA	18.0 ± 3.1	17.3 ± 2.6	0.23
PV area (mm^2^), (mean ± SD)						
LIPV	269.6 ± 220.2	224.1 ± 116.9	0.33	NA	NA	NA
LSPV	281.0 ± 98.4	250.0 ± 94.6	0.08	NA	NA	NA
RSPV	NA	NA	NA	371.6 ± 111.3	312.5 ± 122.7	0.09
RIPV	NA	NA	NA	266.9 ± 88.4	241.8 ± 62.2	0.31
LAWT (mm), (mean ± SD)						
Mean total	1.35 ± 0.46	1.32 ± 0.51	0.78	1.34 ± 0.46	1.32 ± 0.54	0.83
Mean roof	1.39 ± 0.59	1.21 ± 0.53	0.18	1.36 ± 0.56	1.27 ± 0.66	0.52
Mean mid-posterior	1.39 ± 0.60	1.41 ± 0.74	0.91	1.37 ± 0.65	1.45 ± 0.62	0.64
Mean infero-posterior	1.12 ± 0.52	1.09 ± 0.62	0.89	1.09 ± 0.55	1.18 ± 0.57	0.51
Mean left	1.21 ± 0.44	1.18 ± 0.49	0.74	NA	NA	NA
Mean right	NA	NA	NA	1.74 ± 0.66	1.79 ± 0.68	0.74

Significant values are marked in bold

*CT* computer tomography, *LAA flow* left atrial appendage flow, *LA-PV* left atrial-pulmonary veins, *LAWT* left atrial wall thickness, *LIPV* left inferior pulmonary vein, *LSPV* left superior pulmonary vein, *NA* not applicable, *PV diameter* pulmonary vein diameter, *RIPV* right inferior pulmonary vein, *RSPV* right superior pulmonary vein, *SD* standard deviation

### The effect of clinical and CT-derived parameters on the first-pass isolation rate

Complete electrical isolation of all PVs was achieved in 100% of the PVs. No peri-procedural complications occurred. Successful first-pass isolation was achieved in 71 cases on the left side and 67 cases on the right side. Successful first-pass isolation of all PVs was achieved in 51 patients. There were no acute reconnections during the 20 min waiting period after the ablation. Regarding anthropometrics and clinical risk factors, we detected no association with the first-pass isolation success rate, based on univariate regression analysis, regardless of left, right or both sided first-pass isolation. Shorter procedural time was found in those cases, where first-pass isolation was achieved on both sides (p = 0.03).

We also found that LAWT did not influence first-pass isolation rate during PVI guided by our standardized ablation strategy. Among all assessed parameters, only the diameter of the RSPV was associated with the success rate of right-sided first pass isolation, as a wider RSPV diameter led to an easier first-pass isolation (OR 1.01, p = 0.04). Other cardiac CT and echocardiography-derived parameters did not influence the success rate of first-pass isolation (p > 0.05, see Table 4).

**Table 4:** Univariate logistic regression analysis for the detection of predictors of achieving first-pass isolation

Parameters	First-pass isolation in all PVs				First-pass isolation in case of right-sided PVs				First-pass isolation in case of left-sided PVs			
	p value	OR	95% CI		p value	OR	95% CI		p value	OR	95% CI	
Age	0.75	1.00	0.97	1.03	0.18	1.02	0.98	1.06	0.76	1.00	0.97	1.04
Female sex	0.21	0.58	0.25	1.36	0.21	0.51	0.17	1.46	0.77	0.87	0.34	2.18
Body mass index	0.13	1.10	0.97	1.24	0.38	1.06	0.92	1.23	0.49	1.04	0.91	1.20
Diabetes	0.07	0.32	0.09	1.13	0.51	0.65	0.18	2.37	0.14	0.40	0.12	1.35
Hypertension	0.21	1.72	0.73	4.06	0.82	1.11	0.41	3.0	0.23	1.75	0.70	4.38
Hyperlipidaemia	0.02	3.05	1.13	8.20	0.09	3.03	0.81	11.30	0.45	1.48	0.52	4.24
Prior stroke/TIA	0.86	0.83	0.11	6.20	0.94	0.92	0.09	9.38	0.86	1.21	0.12	12.26
Paroxysmal/persistent AF	0.28	1.58	0.68	3.65	0.80	1.12	0.42	2.99	0.75	1.15	0.46	2.87
CHA2DS2-VASc	0.65	0.92	0.66	1.29	0.69	1.08	0.72	1.60	0.28	0.82	0.57	1.17
Ablation parameters												
LAT	0.88	1.00	0.98	1.03	0.99	1.00	0.97	1.03	0.91	1.00	0.97	1.03
Procedure time	0.00	0.95	0.93	0.98	0.16	0.98	0.96	1.00	0.031	0.97	0.94	0.99
Fluoroscopy time	0.21	0.99	0.99	1.00	0.29	0.99	0.99	1.00	0.756	1.00	0.99	1.00
Echocardiographic parameters												
Ejection fraction, (mean ± SD)	0.62	1.01	0.95	1.07	0.21	1.0	0.97	1.10	0.71	0.98	0.92	1.05
LAA flow	0.83	1.00	0.98	1.02	0.20	0.98	0.96	1.00	0.29	1.01	0.98	1.03
CT derived parameters												
LAV, (mean ± SD)	0.60	0.99	0.98	1.00	0.47	0.99	0.98	1.00	0.62	1.00	0.99	1.01
PV diameter, (mean ± SD)												
LIPV	0.29	1.00	0.99	1.00	NA	NA	NA	NA	0.32	1.00	0.99	1.00
LSPV	0.10	1.00	0.99	1.00	NA	NA	NA	NA	0.19	1.00	0.99	1.00
RSPV	0.16	1.00	0.99	1.00	**0.04**	1.00	1.00	1.01	NA	NA	NA	NA
RIPV	0.06	1.00	1.00	1.01	0.22	1.00	0.99	1.01	NA	NA	NA	NA
PV area												
LIPV	0.33	1.04	0.96	1.12	NA	NA	NA	NA	0.33	1.04	0.95	1.15
LSPV	0.12	1.11	0.97	1.27	NA	NA	NA	NA	0.09	1.13	0.98	1.32
RSPV	0.07	1.13	0.99	1.29	0.08	1.15	0.97	1.35	NA	NA	NA	NA
RIPV	0.05	1.15	0.99	1.33	0.30	1.09	0.92	1.29	NA	NA	NA	NA
LAWT, (mean ± SD)												
Mean total	0.35	0.66	0.27	1.59	0.82	1.12	0.40	3.10	0.77	1.15	0.44	2.99
Mean roof	0.56	1.22	0.60	2.49	0.51	1.31	0.56	3.05	0.17	1.74	0.77	3.93
Mean mid-posterior	0.09	0.56	0.28	1.09	0.63	0.83	0.39	1.76	0.90	0.95	0.47	1.93
Mean infero-posterior	0.16	0.58	0.27	1.25	0.50	0.74	0.31	1.78	0.89	1.05	0.46	2.41
Mean left	0.53	1.18	0.68	2.05	NA	NA	NA	NA	0.73	1.18	0.43	3.19
Mean right	0.64	0.88	0.52	1.48	0.73	0.88	0.42	1.83	NA	NA	NA	NA
Mitral isthmus	0.34	0.74	0.40	1.37	0.84	1.07	0.52	2.21	0.67	0.86	0.44	1.70
Left lateral ridge	0.32	1.31	0.76	2.24	0.05	2.01	0.99	4.05	0.93	1.02	0.57	1.84

Significant values are marked in bold

*AF* atrial fibrillation, *CI* confidence interval, *CT* computer tomography, *LAA flow* left atrial appendage flow, *LAT* left atrial time, *LAV* left atrial volume, *LAWT* left atrial wall thickness, *LIPV* left inferior pulmonary vein, *LSPV* left superior pulmonary vein, *PV diameter* pulmonary vein diameter, *RIPV* right inferior pulmonary vein, *RSPV* right superior pulmonary vein, *SD* standard deviation, *OR* odds ratio, *TIA* transient ischemic attack

Reproducibility was assessed in 20 patients at 11 regions of interest (20 × 11 measurements) in terms of wall thickness, moreover the area and diameter of each pulmonary veins were also assessed. The intra- and inter-reader ICC for the assessment of LAWT were 0.98 (CI 0.97–0.98) and 0.92 (CI 0.79–0.97), respectively. The intra-reader area and diameter ICC’s minimum and maximum range were between 0.94 and 0.99 and 0.98–0.99 respectively while the inteR–Reader area and diameter ICC’s minimum and maximum range were between 0.78–0.92 and 0.80–0.94, respectively.

## Discussion

Our main findings indicate that using ablation index with a standardized ablation protocol in drug-refractory AF patients results in a high acute procedural success rate independently from CT-derived LAWT. Regarding the PV anatomy, RSPV diameter might influence the rate of first-pass isolation. The assessment of PV diameters and LAWT were highly reproducible.

Recent advancements in ablation techniques, catheter types and pre-ablation imaging have paved the way for effective and safe therapies in treating AF [10]. A novel ablation quality marker was first introduced by Nakagawa et al., based on a canine study [28]. Ablation index is a quality marker and a surrogate measure for the quality of the ablation lesions [29]. Several studies have reported that it is a useful tool for a durable PVI as it incorporates contact force, power and time in a weighted non-linear formula [29, 30]. In an in vitro study, the reliability of AI was validated with a good correlation with lesion width and lesion depth and volume using different contact angle, RF power and contact force settings [31]. Furthermore, in an in vivo study by El Haddad et al. a substantial difference was found in the minimum value of AI for durable segments between the anterior and posterior parts of the circle, which indicates the role of wall thickness in different regions of the atrium. It has been shown that a higher ablation index value is necessary in the anterior segments to avoid the reconnections [32]. However, data on the optimal ablation index target values on the anterior and posterior wall are controversial [5, 8, 30, 33].

At present, no gold standard measurement for atrial wall thickness is available, however cardiac CT can reliably assess LAWT due to its high spatial and temporal resolution [34–36]. It has been demonstrated that there is an inter-and intra-patient variability in LAWT across paroxysmal versus persistent AF patients [23, 37]. Moreover, several studies investigated the role of LAWT in PVI in light of AF recurrence [38–41]. However, these results are controversial in terms of the locations of the thickest region in LA and the role of LAWT on procedural efficacy [19, 40]. Of note, less advanced ablation techniques were used in these studies.

Mulder et al. first analysed the association between LAWT and acute PV reconnection in those patients who underwent AI-guided AF ablation. Based on their study results, local wall thickness had an impact on the occurrence of acute PV reconnections both in the anterior and posterior segments [14].

These findings are in contrast with our study results as we did not find a connection between LAWT (mean wall thickness of all 11 region) and the acute procedural outcome despite substantial differences across different regions of the LA in our patient population. This discrepancy might be explained by the difference in the procedural endpoints, as we investigated the effect of LAWT on the first pass isolation rate. We could not even use the acute PV reconnection as an endpoint as there was a total absence of acute PV reconnection in our current study using our highly effective, standardized ablation protocol, (e.g. the modified CLOSE protocol) with slightly higher minimal target contact force values than in the study by Mulder et al. (e.g. 10 g vs 5 g) [14], and with the use of a steerable sheath for ablation that might enable more stable catheter-tissue contact during ablations.

Based on the findings of El Haddad et al. this novel approach was introduced for enclosing the PV with optimized and contiguous RF lesions to achieve optimal lesion continuity and depth [5]. The use of these criteria in 130 patients showed a high rate of first-pass isolation [5]. In addition, it proved to be more effective than PVI using only AI with also higher first-pass isolation incidence [7]. Our AI target values (400 on the posterior wall and 500 on the anterior wall) were associated with an acceptably high rate of first-pass PVI isolation and the absence of acute reconnection. Using these AI target values, sufficiently large lesions were created even in case of a thicker atrial wall, indicated by the similar first-pass isolation rates in case of different LAWT values [42]. Thus, our standardized approach seems to be an appropriate choice to create a good quality ablation line that is independent from LAWT. Of note, a recent study showed that LAWT-tailored, individualized AI values might result in similarly good results, even with somewhat lower AI targets. On the other hand, this approach is time consuming, but does not seem to be better in efficacy or safety [43].

Out of all assessed parameters, only RSPV diameter was associated with a higher right-sided successful PVI on first-pass isolation. Despite the fact that the right pulmonary vein region is challenging in terms of PVI, due to its epicardial connection with the carina and the right atrium [44, 45], RSPV diameter size could positively influence the outcome of PVI. In the current study, we demonstrated that wider RSPV diameter could possibly lead to a successful first-pass isolation. This might be explained by the higher freedom in catheter navigation in those cases where the RSPV was not very narrow and thus, the angle between the RSPV and the LA is less pronounced.

Although the results of our study showed that LAWT measured by cardiac CT does not influence the acute success rate of AF ablation using the modified CLOSE protocol, the role of pre-ablation cardiac CT is well established and provides invaluable information for procedural planning, LAA assessment and patient selection. At last, we would like to mention that the procedural safety was excellent with the current standardized ablation protocol, compared to previous results [46].

## Limitations

We acknowledge the limitations of our study. Firstly, this was a single-center study with a relatively low number of patients, however our study provides the first insight into the association of LAWT thickness on the procedural success of first pass ablation using a standardized ablation protocol. On the other hand, LAWT was measured in 11 LA segments in each patient, resulting in a detailed evaluation of the LA identifying its role in contemporary AF management using RF ablation. Although the main focus of the study was the evaluation of the acute procedural success of the modified CLOSE protocol in light of LAWT measurements by cardiac CT, further evaluation of the long-term procedural success (AF recurrence) of the modified CLOSE protocol is warranted. LAV was mostly derived from diastolic phases (due to the clinical CT protocol) and therefore it does not reflect the maximal LA volume for a given patient and limits our conclusions regarding this parameter.

## Conclusion

Using standardized ablation protocol in paroxysmal and persistent AF patients leads to a high first pass isolation rate and high acute procedural success independently from the LAWT. Larger RSPV diameter showed an association with right-sided successful first pass isolation.

## Abbreviations

AF: Atrial fibrillation
BMI: Body mass index
CI: Confidence interval
CT: Computed tomography
ICC: Intraclass correlation coefficient
LA: Left atrium
LA-PV: Left atrium-pulmonary vein
LAWT: Left atrial wall thickness
LIPV: Left inferior pulmonary vein
LSPV: Left superior pulmonary vein
PV: Pulmonary vein
PVI: Pulmonary vein isolation
RIPV: Right inferior pulmonary vein
RSPV: Right superior pulmonary vein
SD: Standard deviation
OR: Odds ratio
TIA: Transient ischemic attack
